# Body mass index and acoustic voice parameters: is there a relationship?^☆^^☆☆^

**DOI:** 10.1016/j.bjorl.2017.04.003

**Published:** 2017-05-06

**Authors:** Lourdes Bernadete Rocha de Souza, Marquiony Marques dos Santos

**Affiliations:** a Universidade Federal do Rio Grande do Norte (UFRN), Departamento de Fonoaudiologia, Natal, RN, Brazil; b Universidade Federal do Rio Grande do Norte (UFRN), Programa de Pós-graduação em Ciências da Saúde, Natal, RN, Brazil

**Keywords:** Body mass index, Obesity, Phonation, Voice, Índice de Massa Corporal, Obesidade, Fonação, Voz

## Abstract

**Introduction:**

Specific elements such as weight and body volume can interfere in voice production and consequently in its acoustic parameters, which is why it is important for the clinician to be aware of these relationships.

**Objective:**

To investigate the relationship between body mass index and the average acoustic voice parameters.

**Methods:**

Observational, cross-sectional descriptive study. The sample consisted of 84 women, aged between 18 and 40 years, an average of 26.83 (±6.88). The subjects were grouped according to body mass index: 19 underweight; 23 normal ranges, 20 overweight and 22 obese and evaluated the fundamental frequency of the sustained vowel [a] and the maximum phonation time of the vowels [a], [i], [u], using PRAAT software. The data were submitted to the Kruskal–Wallis test to verify if there were differences between the groups regarding the study variables. All variables showed statistically significant results and were subjected to non-parametric test Mann–Whitney.

**Results:**

Regarding to the average of the fundamental frequency, there was statistically significant difference between groups with underweight and overweight and obese; normal range and overweight and obese. The average maximum phonation time revealed statistically significant difference between underweight and obese individuals; normal range and obese; overweight and obese.

**Conclusion:**

Body mass index influenced the average fundamental frequency of overweight and obese individuals evaluated in this study. Obesity influenced in reducing maximum phonation time average.

## Resumo

**Introdução:**

Elementos específicos como peso e volume corporal podem interferir na produção da voz e, consequentemente, em seus parâmetros acústicos, razão pela qual é importante que o médico esteja ciente dessas relações.

**Objetivo:**

Investigar a relação entre o índice de massa corporal e os parâmetros acústicos médios da voz.

**Método:**

Estudo observacional, transversal, descritivo. A amostra foi composta por 84 mulheres, com idade entre 18 e 40 anos, média de 26,83 (±6,88). As participantes foram agrupadas de acordo com o índice de massa corporal: 19 abaixo do peso; 23 com intervalos normais, 20 com sobrepeso e 22 obesos e avaliadas quanto a frequência fundamental da vogal sustentada [a] e o tempo máximo de fonação das vogais [a], [i], [u], utilizando o software PRAAT. Os dados obtidos foram submetidos ao teste de Kruskal-Wallis para verificar se houve diferença entre os grupos com relação as variáveis do estudo. Todas as variáveis apresentaram resultados estatisticamente significativos e foram submetidas ao teste não paramétrico de Mann-Whitney.

**Resultados:**

Com relação à média da frequência fundamental, houve diferença estatisticamente significativa entre os grupos com peso normal e sobrepeso e entre os grupos com peso normal e obesidade. A média do tempo máximo de fonação revelou diferença estatisticamente significativa entre indivíduos com baixo peso e obesidade; peso normal e obesidade; sobrepeso e obesidade.

**Conclusão:**

O índice de massa corporal influenciou na média da frequência fundamental dos indivíduos com sobrepeso e obesos avaliados neste estudo. A obesidade mórbida influiu na redução da média do tempo máximo de fonação.

## Introduction

The clinical assessment includes a number of possibilities to evaluate voice production and perception. Among the parameters evaluated in the acoustic analysis of voice are the fundamental frequency and the maximum phonation time (MPT), measurements of interest in voice evaluations.^1^, ^2^

The fundamental frequency (f0), one of the main measures used to characterize the human voice, is directly related to the mass, elasticity and length of the vocal folds, and depends on the subglottic pressure and the configuration of the individual vocal tract.^3^ It provides information about the speaker's characteristics, such as gender, age, emotional state, hormonal competence and body size.^4^, ^5^, ^6^

Several studies seek to understand the influence of body mass index (BMI), muscle mass gain and menopause in acoustic parameters of voice.

D’haeseleer et al.^7^ conducted a study in order to verify the correlation between the BMI and the fundamental frequency of speech in a group of pre and post-menopausal women with and without hormone treatment. The results showed that in post-menopausal women not undergoing hormone treatment increased BMI was correlated with increased f0 of speech. This correlation is explained by the authors through the higher amount of estrogen production in the adipose tissue in women with higher BMI.

In order to analyze the correlation between the acoustic parameters of voice and body height, weight and composition of body mass in young male adults another study was carried out. The results showed no correlation between the composition and distribution of body mass with f0 at the usual pitch.^4^

However, as regards BMI, few studies^1^, ^2^, ^8^ state that body weight interferes with f0 values, which is lower in obese individuals and higher in underweight women.^9^

Another verified measure in voice evaluation is the Maximum Phonation Time (MPT). It is an acoustic, complementary measure, quantified in seconds and used in the diagnosis of patients with dysphonia as well as to check treatment progress. It provides information on respiratory support, glottal efficiency and neuromuscular and aerodynamic balance of voice production.^3^, ^10^

The MPT performance can be influenced by vital capacity and varies according to age, gender and body height and weight.^11^ The MPT values can be impacted in low body weight individuals given their bad performance in lung capacity tests, which can be explained by their physical condition and less muscular firmness.^12^, ^13^, ^14^

Salomon et al.^14^ reported on their studies a weak correlation between lung capacity and MPT, but a strong correlation between larynx airway resistance and MPT. Studies^2^, ^9^, ^15^, ^16^ with the purpose of assessing the MPT in individuals with or without voice changes, regardless of age and sex, should consider the interference of the BMI due to the impact of excessive body weight in abdominal breathing support for voice production.

These measurements, available at a reasonable cost,^17^ have been successful in comparing the data obtained pre and post-speech therapy, thus allowing to understand patient evolution^3^ and therapy efficacy. The gap caused by the lack of studies dedicated to verify the average MPT and the f0 in adults with different BMI motivated this research, which aimed to verify if the BMI interferes in the average of these parameters. It is believed that this study will provide knowledge and subsidies for acoustic evaluation of voice and analysis studies in individuals with higher BMI.

## Methods

### Subjects

Initially, the sample included 99 women, but 27 of them were excluded based on the exclusion/inclusion criteria. At the end, the sample totaled 84 women, aged 18–40, and mean age 26.83 (±6.88). The reason why female subjects were selected for this study lies in the fact that women are more likely to voice changes and more often seek medical/speech care.^18^ Participants were grouped according to the BMI as follows: Group 1 (G1) 19 underweight subjects (BMI below 18.50 kg/m^2^); Group 2 (G2) 23 normal weight subjects (BMI between 18.50 and 24.99 kg/m^2^); Group 3 (G3) 20 overweight subjects (BMI between 25.00 and 29.99 kg/m^2^) and Group 4 (G4) 22 obese individuals (BMI above 30.00 kg/m^2^).^19^ In order to establish the BMI of each participant, all of them were weighed and body mass measured in kilograms using a digital scale. The BMI was calculated as body weight (kg) divided by height (m) and squared. Height was measured using a tape measure. The subject was upright, with arms hanging along the body and heels together, wearing light clothes and barefoot.

### Exclusion criteria

Voice professionals, smokers, individuals with a history of voice disorders and voice quality change from moderate to intense (CAPE-V),^20^ asthma, gastroesophageal reflux, allergies and/or infections of the upper and/or lower respiratory tract and individuals who were in the pre-menstrual period^21^ at the time of data collection. The exclusion criteria were established to avoid confounding factors that could influence voice production and voice quality.

### Methods

The data were collected at the Clinical School of Speech Therapy, in a room with minimal noise −50 db, measured using a decibelimeter iCEL DL40-20. Participants were seated with the microphone 5 cm from their mouth. The fundamental frequency was collected through voice recording using the vowel [a] in usual intensity and pitch for an average period of three seconds in an HP-branded computer with external unidirectional Clone-branded microphone, using program PRAAT^22^ for the analysis and considering sampling range of 44,100 Hz. For the vowel analysis, the beginning and the end of emission were discarded due to phonation instability. In order to check the MPT, each participant was asked to take a deep breath and sustain the vowel as long as possible, in usual intensity and pitch, using the narrowband spectrogram included in the acoustic analysis program to measure the beginning and end of speech with the highest precision.^9^ Vowels [a], [i] and [u] were used in this task. Each participant was asked to sustain the production of each vowel twice, and the best MPT was selected.

### Statistical method

For the analysis of data the measures of central tendency were used to verify data midpoint (median) and data dispersion (percentiles). The data were analyzed using Kruskal–Wallis test for differences between the groups regarding study variables. All variables showed statistically significant results and were submitted to the non-parametric Mann–Whitney test.

This study was approved by the Research Ethics Committee in Human Beings of the institution and complies with all the provisions of Resolution 466/2012 of the National Health Council (CNS), under no. 851,082/14. Participants signed the Free Informed Consent form acknowledging awareness of the confidentiality of the work. Only the quantitative and qualitative information related to this research had value for scientific disclosure purposes.

## Results

The profile of the sample is concentrated in the 18–40 years age group, mean age 26.83, as follows: G1 (22.37 ± 4.57); G2 (22.43 ± 2.35); G3 (29.35 ± 8.71) and G4 (33.00 ± 4.19) and their respective BMI: G1 (17.47 ± 1.16); G2 (22.09 ± 1.59); G3 (28.97 ± 2.47) and G4 (44.88 ± 7.38). Table 1 shows that the average value of f0 was higher in G2. G3 showed the lowest average for this variable. Although the amplitude difference of G1 and G2 medians is 11 Hz, this difference was not significant (Table 2). As for G3 and G4, the values between medians showed up very close. The average MPT was higher in G2 in vowel [i], and lower in G4 in vowel [a]. It is noteworthy to highlight that the group of obese individuals (G4) showed average MPT lower than 10 s limit.^11^ G2 showed higher f0 with statistically significant results when compared to G3 and G4 (Table 2).

**Table 1:** Average and standard deviation of age, f0 and MPT of the groups assessed as to BMI.

Variables	Age (years) Average SD	f0 (Hz) Average SD	MPT (s)		
			Vowel [a] Average SD	Vowel [i] Average SD	Vowel [u] Average SD
(G1) Underweight	22.37 ± 4.57	213.79 ± 24.67	11.42 ± 2.77	12.63 ± 3.20	13.16 ± 3.04
(G2) Normal range	22.43 ± 2.35	223.48 ± 16.79	12.48 ± 2.69	13.00 ± 3.42	12.70 ± 2.70
(G3) Overweight	29.35 ± 8.71	206.09 ± 27.07	11.45 ± 2.59	11.35 ± 2.25	11.70 ± 2.31
(G4) Obese	33.0 ± 4.19	208.55 ± 24.32	9.27 ± 1.90	9.91 ± 3.36	9.41 ± 3.34

s, seconds; f0, fundamental frequency, MPT, maximum phonation time; BMI, body mass index.

**Table 2:** Average values and percentiles Q25 and Q75 and *p*-value of the assessed groups as age, f0 and MPT.

	Group	Median	Q25–Q75	*p*-Value
Age	1	21.00^a^	20.00–22.00	<0.001
	2	22.00^a^	21.00–23.00	
	3	27.50^b^	22.00–39.75	
	4	33.00^b^	28.75–35.00	

f0	1	211.00^a^	193.00–235.00	0.033
	2	222.00^a^	211.00–230.00	
	3	200.00^b^	187.50–217.00	
	4	195.50^b^	191.75–226.50	

MPT [a]	1	11.00^a^	9.00–13.00	0.001
	2	12.00^a^	10.00–14.00	
	3	11.50^a^	9.00–13.75	
	4	9.00^b^	9.00–10.25	

MPT [i]	1	12.50^a^	10.00–16.00	0.012
	2	12.00^a^	11.00–15.00	
	3	11.00^a^, ^b^	10.00–13.00	
	4	9.00^b^	7.75–12.25	

MPT [u]	1	13.00^a^	11.00–16.00	0.001
	2	12.00^a^	10.00–15.00	
	3	11.50^a^	10.00–13.00	
	4	8.50^b^	7.00–12.25	

f0, fundamental frequency; MPT, maximum phonation time.

Kruskal–Wallis test, *p* < 0.05.

a No statistically significant difference.

b With a statistically significant difference.

## Discussion

The objective of this study was to investigate if the fundamental frequency of voice and the maximum phonation time could be influenced by body weight when analyzing the average of these parameters into four groups of females with different BMI.

Importantly, despite the statistically significant difference between the groups as regards age (Table 1), this variable was not relevant because all participants belong to the same period of greatest vocal efficiency.

The relation between body weight and voice has been subject of discussion in some studies, suggesting that obese individuals are expected to present different vocal characteristics.^1^, ^2^, ^8^ However, the literature is scarce in evaluating these parameters in subjects with different BMI.

One of the main acoustics components of voice, the fundamental frequency, provides information about the speaker's physical attributes such as gender, age, emotional state, hormonal competence and body size.^4^, ^5^, ^6^

Studies^23^ report that acoustic track of voice may be closely related to body size. However, Titze^24^ shows that vocal fold length, biomechanical stress, and laryngeal muscle activity are the main factors responsible for f0 change.

G2 had an average f0 higher than G1, but this difference was not statistically significant. This result differs from another study that found higher average f0 in individuals with low weight and significant difference between low weight and normal weight individuals,^9^ and supports Titze^24^ statement by arguing that vocal fold length, biomechanical stress and laryngeal muscle activity are the main variables responsible for f0 change.

There was statistically significant difference between G1 and G3/G4, and between G2 and G3/G4 as regards this variable, which corroborates studies that show average of fundamental frequency for female pattern in obese women.^1^, ^2^, ^24^ A possible explanation for this difference between the groups is the interference of excessive body weight in abdominal breathing support for voice production,^2^, ^9^, ^15^, ^16^ driving to increased laryngeal muscle activity,^12^ thus resulting in the reduction of this parameter value.

The maximum phonation time is obtained by the interaction between total capacity of air available for voice production, expiratory force and adjustment of the larynx for the efficient use of air, i.e., glottal adduction and resistance.^3^ This is the simplest aerodynamic parameter and one of the most commonly used measures in clinical evaluation of voice.^18^ In this study, the average of this variable showed statistically significant difference between G4 and the other groups.

Underweight individuals (G1) showed higher average MPT compared to overweight (G3) and obese individuals (G4) (Table 2). Studies^12^, ^13^ report that this parameter can be impacted in underweight individuals due to the poor performance observed in their lung function. Our results did not show this hypothesis.

G1, G2 and G3 showed higher average MPT compared to obese individuals with statistically significant difference (Table 2). This result may be due to increased subglottic pressure found in individuals with higher body weight and explain the need to overcome the effects of increased pharyngeal resistance in these individuals.^9^ This effect may have contributed to phonation effort and reduced the average MPT among obese individuals, explaining the difference found between the groups.

The reduction in lung function due to increased adipose tissue around the ribs and abdomen drives to reduced chest compliance and respiratory muscle strength capacity. This is based on the fact that overweight increases tissue mass in the neck, chest and upper airways, particularly the larynx,^25^ and may have determined the difference found between the groups regarding this task.

There was statistically significant difference between G1/G2/G3 and G4 (Table 2). This can also be explained by the interference of weight gain in abdominal breathing support during this task,^16^ which favored the reduction of this parameter average. This result is consistent with authors^14^ that show a weak correlation between lung capacity and MPT, but a strong correlation between larynx airway resistance and MPT. This assumption is based on results and findings of other studies, because lung capacity and vocal tract diameter measures were not investigated in our study.

G4 was the group that showed lower MPT average on vowels [a] and [u] compared to other groups, with statistically significant difference. This difference may be explained by the interference of reduced chemoreceptor sensitivity in obese individuals, which is offset by the hyperactivity of the pharyngeal dilator muscles,^26^ thus reducing the sustained emission of these vowels.

No significant differences were found between G3 and G4 as regards the MPT of vowel [i] (Table 2). This result may reflect the characteristics of this vowel. As a tense vowel, it may require greater vocal effort, the need to overcome the effects of pharyngeal resistance^27^ and reduced average MPT in individuals with higher body weight.

This study sought to collaborate with the literature and provided preliminary results on acoustic measures of voice in women with different BMI. Based on study results, it is suggested that during the evaluation of MPT measures the BMI of overweight and obese individuals is also taken into account.

### Study limitations

This study had some limitations that were not possible to solve. The number of participants could have been higher, but the exclusion criterion to avoid confusing factors reduced the sample size. Future studies could provide subsidies in order to obtain reliable information about the endoscopic evaluation of the vocal tract diameter of women with different BMI and the videolaryngoscopic assessment. The access to these techniques, though, is limited and expensive.

## Conclusion

The results suggest that the reduced f0 values were influenced by body weight in overweight and obese individuals.

Obese women showed lower MPT, which may have been influenced by the increased adipose tissue around the ribs and abdomen, favoring a strong reduction of this parameter.

The results of this study showed that, although the anatomical elements are identical among the individuals, they do not present the same physiological characteristics, reason why the understanding of body weight influence in the acoustic parameters of voice during vocal evaluation is a relevant task to be considered.

## Conflicts of interest

The authors declare no conflicts of interest.
